# High-Temperature, Lightweight Ceramics with Nano-Sized Ferrites for EMI Shielding: Synthesis, Characterisation, and Potential Applications

**DOI:** 10.3390/ma16247615

**Published:** 2023-12-12

**Authors:** Vitalijs Abramovskis, Ilmars Zalite, Mikhail Maiorov, Janis Baronins, Ashish Kumar Singh, Vjaceslavs Lapkovskis, Saurav Goel, Andrei Shishkin

**Affiliations:** 1 Laboratory of Ecological Solutions and Sustainable Development of Materials, Institute of General Chemical Engineering, Faculty of Materials Science and Applied Chemistry, Riga Technical University, Pulka 3, K-3, LV-1007 Riga, Latvia; vitalijs.abramovskis@edu.rtu.lv (V.A.); janis.baronins@rtu.lv (J.B.); vjaceslavs.lapkovskis@rtu.lv (V.L.); 2 Institute of Materials and Surface Technologies, Riga Technical University, P. Valdena Iela 7, LV-1048 Riga, Latvia; ilmars.zalite@rtu.lv; 3 Institute of Physics, University of Latvia, Miera Iela 32, LV-2169 Salaspils, Latvia; maiorov@sal.lv; 4 SMW Group AS, Kr. Barona Street 3-1, LV-1050 Riga, Latvia; aksingh@smw.com; 5 School of Engineering, London South Bank University, London SE1 0AA, UK; goels@lsbu.ac.uk; 6 Department of Mechanical Engineering, University of Petroleum and Energy Studies, Dehradun 248007, India

**Keywords:** cenospheres, CoFe_2_O_4_, high-temperature sintering, magnetic properties, syntactic foam

## Abstract

The present study focuses on the synthesis and characterisation of a lightweight ceramic material with electromagnetic interference (EMI) shielding properties, achieved using mullite containing micrometre-sized hollow spheres (cenospheres) and CoFe_2_O_4_ nanoparticles. This research explores compositions with varying CoFe_2_O_4_ contents ranging from 0 up to 20 wt.%. Conventional sintering in an air atmosphere is carried out at a temperature between 1100 and 1300 °C. The addition of ferrite nanoparticles was found to enhance the process of sintering cenospheres, resulting in improved material density and mechanical properties. Furthermore, this study reveals a direct correlation between the concentration of ferrite nanoparticles and the electromagnetic properties of the material. By increasing the concentration of ferrite nanoparticles, the electromagnetic shielding effect of the material (saturation magnetisation (*M*_s_) and remanent magnetisation (*M*_r_)) was observed to strengthen. These findings provide valuable insights into designing and developing lightweight ceramic materials with enhanced electromagnetic shielding capabilities. The synthesized ceramic material holds promise for various applications that require effective electromagnetic shielding, such as in the electronics, telecommunications, and aerospace industries.

## 1. Introduction

Radiation fields from electronic devices like antennas, phones, and household appliances cause electromagnetic interference (EMI). Shielding sensitive electronics from EMI is challenging for aircraft, military, and communication system components [1]. Efforts to combat EMI involve developing shielding systems for intentional or unintentional interference. However, using metallic materials for EMI shielding is becoming difficult due to the trend toward smaller and lighter electronic packaging [2].

Industry traditionally uses electrically conductive metals for EMI shielding, but their high density and susceptibility to corrosion lead to heavy shielding components. To address this, researchers have turned to low-density materials like polymers, which have poor electrical conductivity and EMI shielding abilities. Two approaches are used to prepare polymer shields: coating with conductive metals or blending with conductive fibres and particles [3]. Researchers have attempted to reinforce transparent polymers with conductive fillers like carbon nanofibers [4,5], carbon nanotubes [6], graphene [7,8], and metallic nanowires and particles [9,10]. Syntactic foams are lightweight composite materials with hollow particles dispersed in a polymer matrix that offer weight-saving potential and are being explored for their EMI shielding effectiveness [11].

The development of lightweight and mechanically robust porous or cellular materials is one of the current trends in modern materials design. In recent years, the use of cenospheres (CSs) for manufacturing porous, lightweight composite materials has been extensively investigated. CSs are three-dimensional objects with a spherical shape that contain a cavity or empty spaces inside. These materials are chemically multicomponent systems with an SiO_2_-Al_2_O_3_-Fe_2_O_3_ content of about 90 wt.% [12,13,14]. Due to their low apparent density (0.40–0.72 g·cm^−3^), low thermal conductivity (about 0.065 W·m^−1^·K^−1^), and excellent stability in alkaline solutions and at high temperatures, cenospheres are promising raw materials for the development of new porous materials. The particle size of cenospheres ranges from about 40 up to 500 µm [15,16].

The civil engineering industry widely applies CSs in producing lightweight concrete with improved thermal insulation [14,17]. CSs also adopt filler and reinforcement roles in metal–matrix [18,19,20] and polymer–matrix [20,21] composites. However, researchers rarely report results of CSs containing low-density ceramic–matrix composites. However, interest in ceramic–cement composites has increased in recent years, and researchers have reported some stunning results.

CSs exhibit reliable sintering tendencies while maintaining their spherical shape, yet they do not possess the ability to form durable structures. Therefore, adding a second phase which promotes the adhesion of the material can improve its mechanical strength. In addition, various additives can promote sintering, e.g., metals, oxides, or other additives. For example, one way to enhance sintering could be the use of oxides, e.g., ferrite nanoparticles [22].

Cobalt ferrite (CoFe_2_O_4_) has excellent chemical stability, mechanical hardness, and electrical insulation. On the other hand, it is a hard magnetic material, and its magnetic properties exhibit size dependence. CoF_2_O_4_ can be synthesised via the sol–gel method with subsequent auto combustion [23], reverse coprecipitation [24], and direct and hydrothermal synthesis [25]. CoF_2_O_4_ has broad applications in various fields [26], from their use as antimicrobial components, in which superparamagnetic cobalt ferrite NPs are used against several kinds of pathogenic microorganisms [27], to the design of EMI shielding materials [28,29,30]. Therefore, using CoFe_2_O_4_ nanoparticles (NPs) in composite production could have a positive effect. In addition, the spinel structure of CoFe_2_O_4_ allows for the introduction of different metallic ions into its lattice, thus altering its structural, magnetic, electrical, and catalytic properties [31]. It has many applications, such as in magnetic data storage, hybrid electric vehicles, transformer cores, high-frequency integrated inductors, biocompatible magnetic fluids, magnetic resonance imaging, and controlled drug delivery. Other industries also implement CoFe_2_O_4_ in manufacturing microwave-absorbing paints, catalysis, hybrid supercapacitors, and products for different applications [32,33]. Remarkable magnetic properties, such as high coercivity (*H*_c_), the magnetocrystalline anisotropy constant (K), Curie temperature (*T*_c_), moderate saturation magnetisation (*M*_s_) and remanent magnetisation (*M*_r_), a high magnetostriction coefficient (λ), and low eddy current losses, along with excellent chemical and mechanical stability, electrical resistance, optical and dielectric properties, low toxicity [34,35,36,37], and impressive mechanical hardness, present CoFe_2_O_4_, as a good candidate for producing syntactic foams with CoFe_2_O_4_.

CoFe_2_O_4_ nanoparticles have been used as standalone materials and incorporated into other materials, such as polymers and carbon-based materials, to improve their magnetic properties. Significant studies in the literature describe the various material design methods and applications of these materials, including EMI shielding, catalysts, and magnetism-related applications [38,39,40,41,42,43,44,45]. Spinel CoFe_2_O_4_ prepared via sol–gel-assisted sintering at 1150 °C by Caldeira et al. [46] exhibited a Vicker’s hardness value of 133.9. Hollow CoFe_2_O_4_ spheres on a carbon template were prepared for high wave absorption by Zhou et al. [47]. A porous, lightweight nanocomposite of CoFe_2_O_4_ and graphene oxide was prepared by Liu et al. [48] that displayed high microwave absorption [31].

For use in real-life EMI shielding applications, a material must have sufficient mechanical strength and a low density. The aerospace industry has an additional requirement for the materials—they must withstand high temperatures (up to 1000 °C). But most the EMI shielding materials are mainly made of (or contain) polymer materials [28], which decompose at 250–350 °C, or include graphite or graphene [28], which burn out at 350–450 °C. 

In this study, a preliminary investigation into the formability and structural integrity of a novel CS-CoFe_2_O_4_ composite foam is conducted. With the incorporation of CoFe_2_O_4_ nanoparticles into a CS matrix, a lightweight, high-temperature ceramic material capable of electromagnetic wave absorption can be produced. Our study focuses on a concentration of 20 wt% CoFe_2_O_4_ nanoparticles. Nevertheless, we anticipate that the enhancement in EMI shielding performance could be further proportional to the ferrite concentration. It is essential to consider that as the ferrite concentration increases, the specific weight of the material also increases, thereby imposing limitations on the material’s potential applications. Generally, a high-performance absorber must possess strong absorptivity, a low density, a broad absorption frequency, and a thin thickness to satisfy the requirements of actual applications [49,50,51]. The effects of sintering temperature and compaction pressure (CP) on mechanical properties were investigated. This article also demonstrates the effect of CoFe_2_O_4_ nanoparticles as an additive for the sintering of CSs while improving magnetic properties [32,33]. The authors study two ceramic types: pure CSs and CS-based ceramics with the addition of CoFe_2_O_4_. The following characterisation methods, materials, and sintering approach demonstrate the effects of the applied compressive pressure, sintering temperature, and the amount of added ferrite on the density and properties of ceramics. This paper investigates and discusses the material’s mechanical strength, porosity, and density. Based on these observations, the recipe with the best combination of properties will be used in further EMI absorber design and study.

## 2. Materials and Methods

### 2.1. Materials

The authors selected mullite-based CSs (Biotecha SIA, Riga, Latvia) [15] with two different particle size distributions, 63–150 μm (CS2) and 150–250 μm (CS1). The authors provided a more detailed description of selected CS properties in one of their previous publications [15]. 

High-frequency plasma chemical synthesis-prepared CoFe_2_O_4_ nano powder was obtained using the chemical co-precipitation method [52]. It had an average particle size of 40 nm [53]. A phase diagram of the ferrite is represented by XRD in the Results and Discussion sections.

### 2.2. Sintering Process

**Figure 1:**
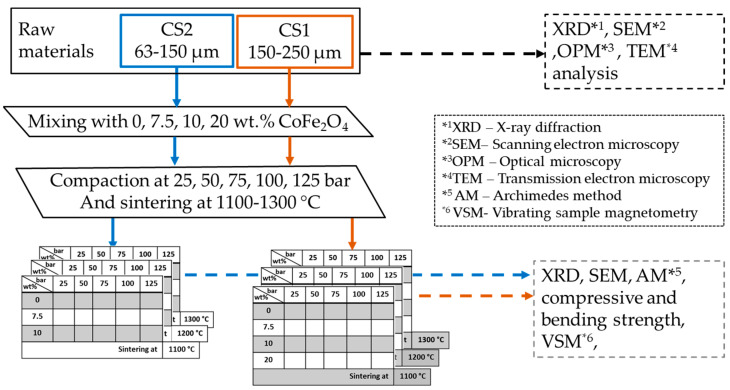
Flowchart of receiving and researching CoFe_2_O_4_ composite material.

A flowchart of the methodology used for this research is shown in Figure 1. Sintering was performed at 1100–1300 °C in an LHT-08/18 furnace (Nabertherm GmbH, Lilienthal, Germany) in an air atmosphere with a heating rate of 10 °C·min^−1^ and a residence time at the maximum temperature of 1 h. The CP of the samples was 25–125 bar. Cylindrical sample size ᴓ = 12 mm and h = 10 mm. Samples measuring 65 × 65 × 9 mm were made to determine the mechanical properties of the material.

### 2.3. Applied Characterization Methods

An operator employed an Advance D8 Bruker AXS (Bruker GmbH, Karlsruhe, Germany) X-ray diffraction (XRD) analysis system with Cu-Ka radiation, utilising the ICDD database PDF-4+2008, PDF-4/Organics 2008, and Sleve+2008 software Version 4, to characterise the phase compositions of the raw materials and sintering products. An operator used Hitachi S4800 (Hitachi High-Tech Europe GmbH, Krefeld, Germany) and Mira/Tescan (TESCAN GmbH, Dortmund, Germany) scanning electron microscopes (SEMs) to evaluate the morphology and microstructure of the CSs and the produced ceramic composites. In addition, researchers conducted optical imaging using a Keyence VHX-2000 digital optical microscope (Keyence Ltd., Osaka, Japan) equipped with Keyence VH-Z20R/W and VH-Z500R/W lenses.

Furthermore, the TEM JEM-100S (JEOL Ltd., Tokyo, Japan) was employed to analyse the nanoparticles’ microstructure. The laboratory assistant used the Archimedes method to determine the bulk density, apparent porosity, water absorption, and apparent specific gravity of the sintered samples. The technician conducted compressive and bending strength tests using a ToniNorm Tinius Olsen 25ST apparatus (Tinius Olsen Inc., Horsham, PA, USA). The magnetic properties of the manufactured materials were analysed using vibrating sample magnetometry (VSM) with a Lake Shore Cryotronics Inc. (Tinius Olsen Inc., Westerville, OH, USA) device, model 7404 VSM.

Apparent density and porosity were determined using the Archimedes method at 20 °C, using distilled water as a liquid medium for immersion. 

## 3. Results and Discussion

**Figure 2:**
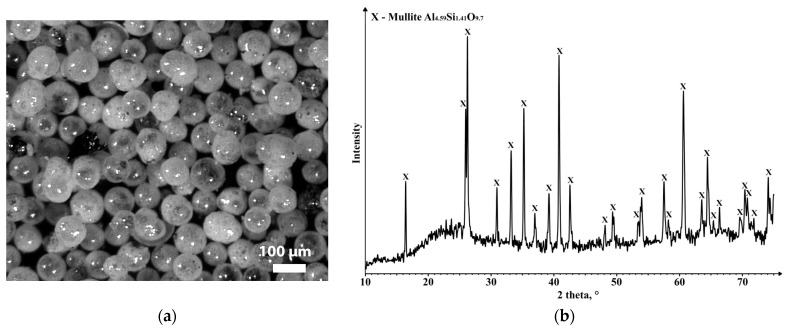
Images of (**a**) the CS2 sample obtained (optical microscope) and (**b**) the phase composition of mullite containing cenospheres with diameters from 63 to 100 μm (sample CS1 exhibited a similar result).

The mullite-based CSs exhibit exceptional mineral mechanical strength and chemical corrosion resistance. Their robustness makes them ideal for applications requiring durability and resilience in high-temperature and corrosive environments. The specific sintering temperature and pressure needed to maintain the hollow nature of the mullite-based CSs, and sintering them into a single object can vary depending on various factors, including the composition of the cenospheres and the desired final properties. However, generally, a typical sintering temperature range for mullite-based CSs is around 1200 to 1600 °C. The pressure applied during the sintering process can vary, but the process is often conducted under atmospheric pressure or in a low-pressure environment to avoid collapsing the hollow structure. An optical image and the representative XRD spectrum of the received CS2 are shown in Figure 2a and Figure 2b, respectively.

**Figure 3:**
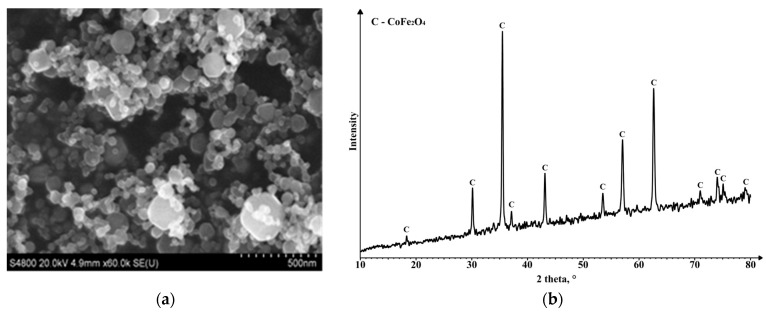
Images of (**a**) CoFe_2_O_4_ nanoparticles obtained via plasma chemical synthesis (SEM) and (**b**) the phase composition of the CoFe_2_O_4_ nano powder (d_50_ = 40 nm).

The CoFe_2_O_4_ nanopowder contained different sizes (Figure 3a), and its XRD spectrum (Figure 3b) matches the peaks of CoFe_2_O_4_. 

**Figure 4:**
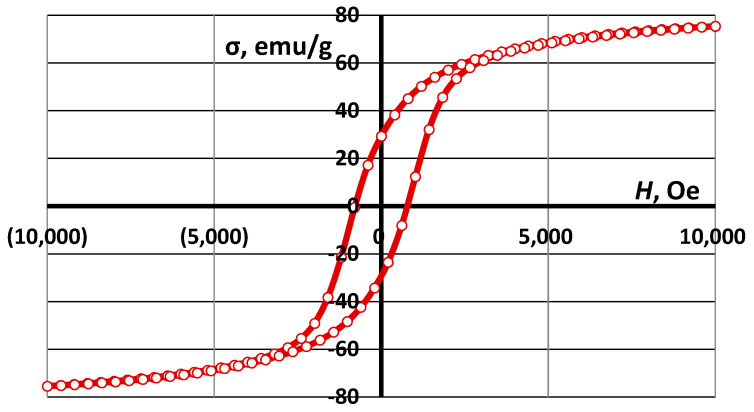
Magnetic properties of CoFe_2_O_4_ ferrites synthesised in plasma.

These nanoparticles can be sintered completely at 1100–1200 °C [54], forming a compacted material. The electromagnetic properties of the pure CoFe_2_O_4_ material are shown in Figure 4 and Table 1. A small addition of nanoparticles can create a connective network between the cenospheres, improving the strength of the structure. The compositions with CoFe_2_O_4_ contents ranging from 0 to 20 wt.%. Therefore, the authors expect a lowered sintering temperature to produce CS-based ceramic materials with CoFe_2_O_4_ nanoparticle additives [15].

Most of the studies were carried out using CS1 cenospheres with a sphere size in the range of 150–250 μm, as well as using CS2 cenospheres. The results show that as the sintering temperature increases, the density of the material increases due to a decrease in moisture content and apparent porosity due to the consolidation of material. A similar trend was observed with an increasing CP used to produce samples. In all sintering modes, regardless of the size of the ferrite nanoparticle additive, as the CP of the samples increases, the density of the material increases, and the apparent porosity of the material decreases. The density of a cellular material has a positive impact on its mechanical response. A similar relationship between density and compressive strength was observed in this study as well. Various concentrations of ferrites were used in the composite material: 0 wt. % (CS1-0, CS2-0), 7.5 wt. % (CS1-7.5 and CS2-7.5), 10 wt. % (CS1-10, CS2-10), and 20 wt. % (CS1-20). In all these variants, it was seen that as the concentration of ferrite nanoparticles increased, the density of the material increased irrespective of the sintering temperature. With increasing density (decreasing porosity) the compressive strength of the materials also increases.

For the range of sintering parameters used in the study (*p* = 50 bar to 125 bar, t = 1100 °C to 1300 °C, and CoFe_2_O_4_ content from 0 to 20 wt.%), samples with a density of 1.19–2.16 g·cm^−3^ and a compressive strength of 1.5–125 MPa were obtained.

**Figure 5:**
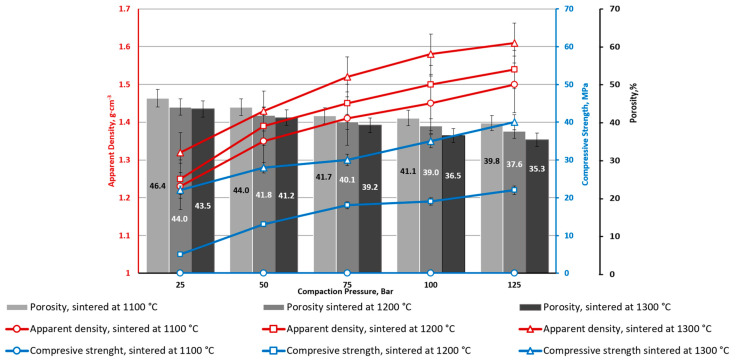
Ceramics’ apparent density, apparent porosity and compressive strength depend on CP and sintering temperature (CS2-0).

Composition CS2-0 (without CoFe_2_O_4_), sintered at 1100 °C and compacted at all studied CP ranges (25–125 Bar), has very low strength (below 1 MPa) (Figure 5).

**Figure 6:**
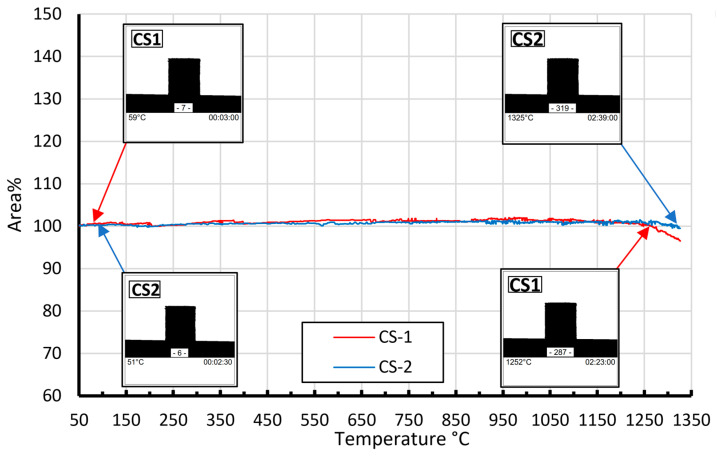
CS1 and CS2 high-temperature optical dilatometry curves in the 50–1325 °C temperature interval, adopted from [15]. Reprint on CC BY, 4.0 Open Access licence from [15].

This fact could be explained by the low adhesion of CS particles to each other due to the low (1100 °C) temperature. As described in previous research [15], this type of CS has good thermal stability (no softening; no shrinkage below 1250 °C). This is clearly seen in Figure 6, where the observed area’s cross-section of CS1 decreases starting from 1250 °C.

The identical weak particles’ adhesion to each other was noticed for specimens sintered at 1200 °C and CP 25 bar. At the same time, other series of specimens sintered at 1300 °C (CP = 25 ÷ 125 bar) and 1200 °C (except CP = 25 bar) demonstrate significant enough mechanical strength. As shown in Figure 5, increasing the sintering temperature from 1100 to 1200 °C resulted in an in average compressive strength increase of 20 MPa. Interestingly, the density of the CS2-0 sintered materials increases gradually (and porosity has reverse dependence, which is logical but also occurs gradually) for all studied sintering temperatures and CP values. The mechanical properties increase drastically by increasing the sintering temperature from 1100 to 1200 and 1300 °C—an average increase of 20 MPa per 100 °C. This could be explained by the CSs softening as a result of better compaction, which is also supported by high-temperature optical dilatometry curves (Figure 6). However, the CP makes a much smaller contribution to the mechanical properties of the samples CS2-0 when compared with the sintering temperature: 2–3 MPa for 1200 °C and 3–6 MPa for 1300 °C.

**Figure 7:**
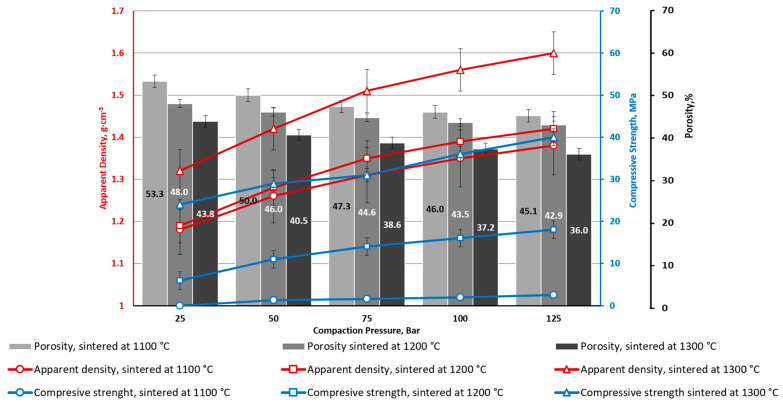
Ceramic apparent density, apparent porosity, and compressive strength depending on CP and sintering temperature for CS1-0.

Composition CS1-0 demonstrates quite similar dependences but demonstrates a slightly higher compression strength in the series sintered at 1100 °C than the same series of CS2-0. Sintered at 1100 °C and compacted at all studied CP ranges (25–125 bar), it has meagre strength (below 3 MPa) (Figure 7). The identical weak particles’ adhesion to each other was noticed for specimens sintered at 1200 °C and a CP of 25 bar. At the same time, other specimen series sintered at 1300 °C (CP = 25 ÷ 125 bar) and 1200 °C (except CP = 25 bar) demonstrate significant enough mechanical strength.

**Figure 8:**
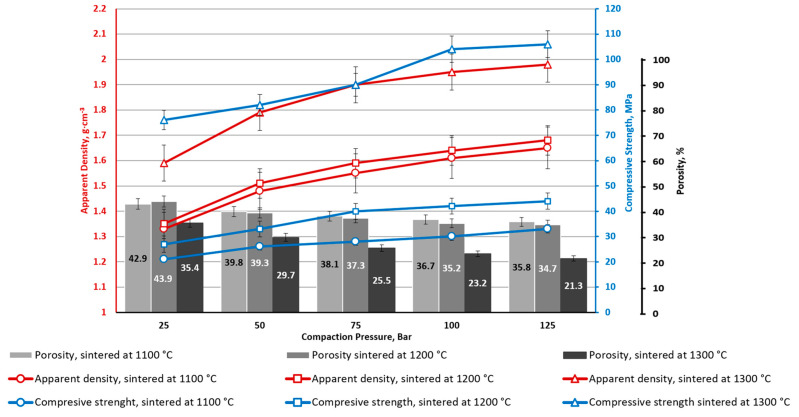
CS1-7.5 porous ceramics with 7.5 wt. % CoFe_2_O_4_: apparent density, apparent porosity, and compressive strength depending on CP and sintering temperature.

**Figure 9:**
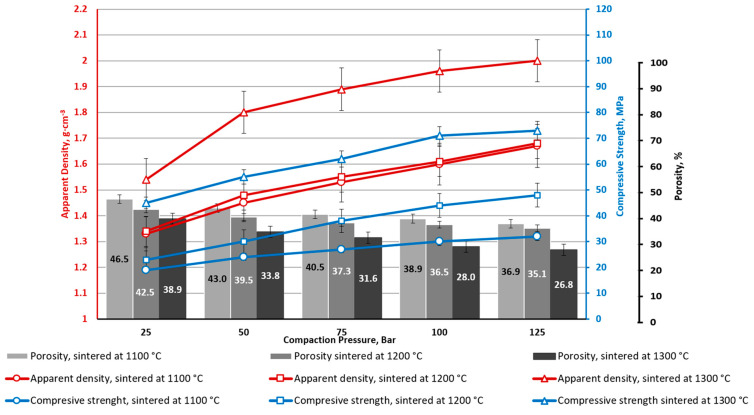
CS2-7.5 porous ceramics with 7.5 wt. % CoFe_2_O_4_: apparent density, apparent porosity, and compressive strength depending on CP and sintering temperature.

Series with CoFe_2_O_4_ have lower average porosity values of 10% (for CS1) and 7% (for CS2) (Figure 8 and Figure 9). This is obvious due to interparticle voids filling with CoFe_2_O_4_ NPs. Filling the voids with NPs also leads to an increase in the contact point spots of the CS particles, which increases the material’s mechanical properties. Specimen series CS1-7.5 and CS2-7.5 (with 7.5 wt.% of CoFe_2_O_4_), at all studied CP and sintering temperature ranges, are characterised by a higher average compressive strength for 30 MPa in comparison to the material without the CoFe_2_O_4_ addition. The overall tendency of the influence of the CP on compressive strength is the same as for CSs without NPs: it gradually increases.

**Figure 10:**
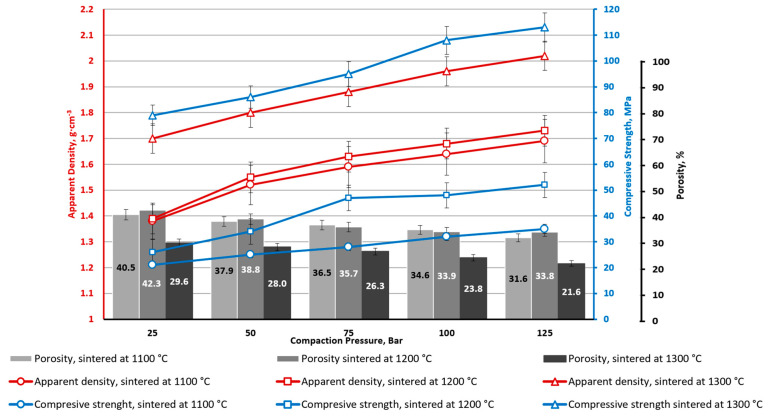
CS1-10 porous ceramics with 10 wt.% CoFe_2_O_4_: bulk density, apparent porosity, and compressive strength depending on CP and sintering temperature.

**Figure 11:**
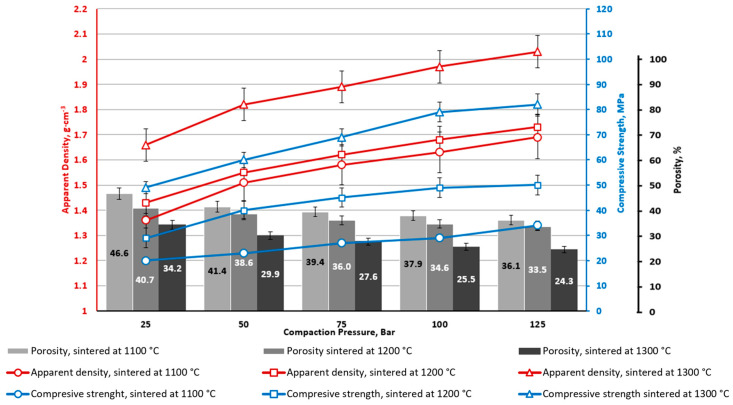
CS2-10 porous ceramics with 10 wt.% CoFe_2_O_4_: bulk density, apparent porosity, and compressive strength depending on CP and sintering temperature.

Similar regularities are also determined for materials with a ferrite content of 10 wt%. (Figure 10 and Figure 11). Comparing the results obtained using cenospheres with a size of 150–250 μm (CS1) and 63–150 μm (CS2), it can be concluded that the size of the cenospheres does not significantly affect the sintering results—the bulk density and apparent porosity. As is known from [15], at temperatures above 1200 °C, cenospheres begin to deform (Figure 6) plastically, which can explain the significant increase in sample density for samples sintered at 1300 °C compared to samples sintered at temperatures of 1100 or 1200 °C. Plastic deformation leads to the material’s consolidation by reducing interparticle voids without the particle breaking.

**Figure 12:**
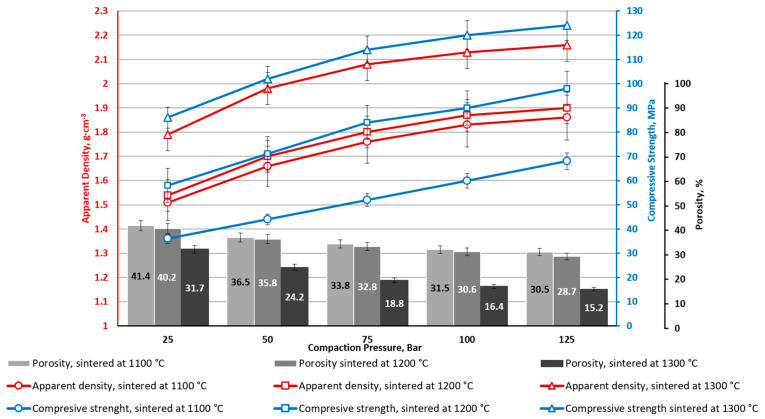
CS1-20 ceramics with 20 wt.% CoFe_2_O_4_ bulk density: apparent porosity and compressive strength depending on CP and sintering temperature.

The nature of the samples with 20% ferrite additives (Figure 12) is similar to the samples with a smaller amount of ferrite additives: the density and compressive strength of the samples increases with increases in the pressing pressure and sintering temperature. Greater ferrite addition increases both material density and compressive strength.

**Figure 13:**
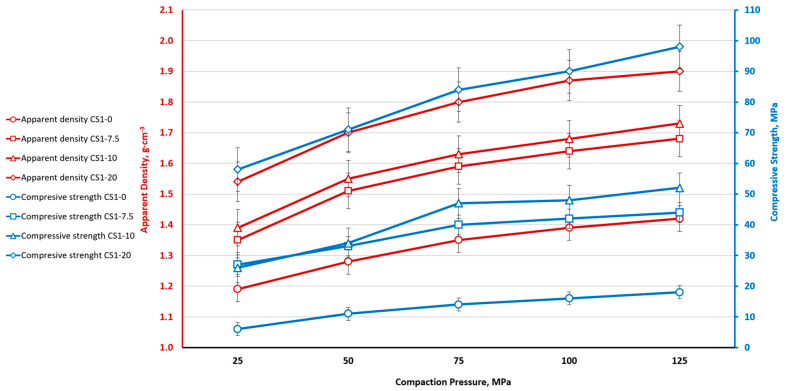
Effect of CoFe_2_O_4_ concentration on the apparent density and compressive strength of cenosphere CS1 ceramics (sintering temperature: 1200 °C).

The relationship between ferrite NP content, CP, apparent density, and compressive strength for CS1 is shown in Figure 13. There is a clear relationship between the material’s apparent density (red curves), compressive strength (blue curves), and ferrite concentration—with an increase in ferrite concentration, the apparent density and compressive strength also increase. Apparent density and the increase in compressive strength increase monotonically as CP increases.

**Figure 14:**
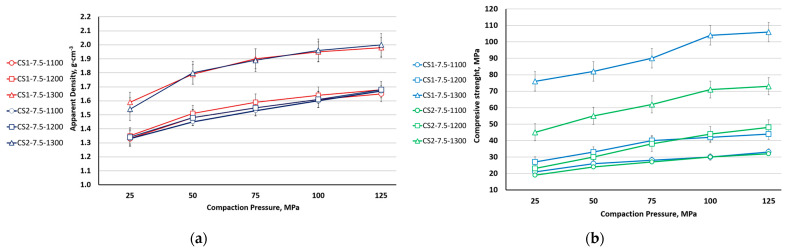
Ceramic relative density (**a**) and compressive strength (**b**) depending on CP and sintering temperature for CS1-7.5 and CS2-7.5.

The relationship between ceramic apparent density (a), compressive strength (b), and CP and sintering temperature for CS1-7.5 and CS2-7.5 is shown in Figure 14. As can be seen from Figure 14, the density of the samples grows monotonically with increasing com-paction pressure and does not depend on the size of the used cenospheres. In contrast to samples sintered at 1100 and 1200 °C, the density of the samples sintered at 1300 °C is significantly higher, which could be related to the pronounced plastic deformation of the cenospheres at this temperature. On the other hand, the compressive strength of the samples sintered at 1300 °C is significantly higher for the CS1 (150–250 μm) samples than for CS2 (63–150 μm) samples. 

**Figure 15:**
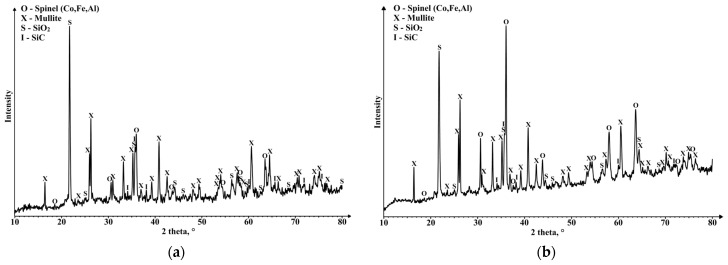
Phase compositions of ceramics: (**a**) CS1–10 sintered at 1200 °C and (**b**) CS1-20 sintered at 1300 °C.

An analysis of X-ray phases (Figure 15) shows that in the case of cenospheres and CoFe_2_O_4_ ceramics, their partial interaction takes place—the mullite partially decomposes and Al_2_O_3_ enters the structure of the CoFe_2_O_4_, while SiO_2_ precipitates as a separate phase. The presence of the SiO_2_ phase in sintered samples can also be partly explained by the crystallisation of amorphous SiO_2_ on the cenospheres.

**Figure 16:**
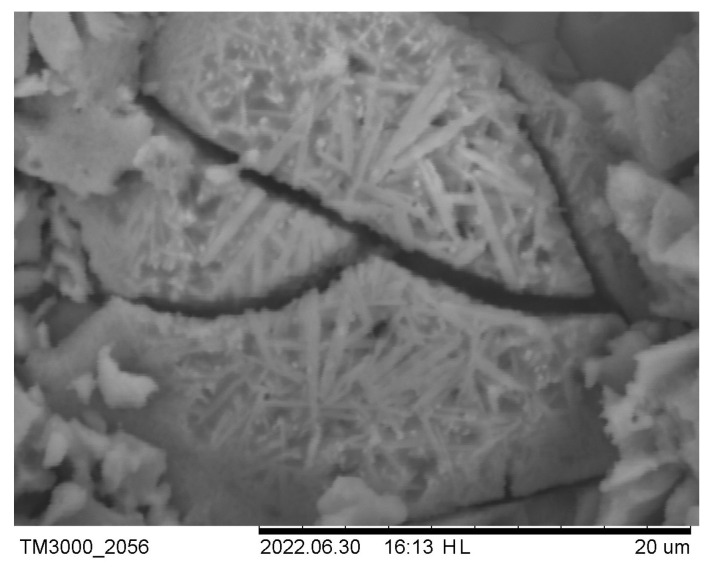
At a temperature of 1200 °C, sintered CS1-0 cenospheres (CP 250 bar).

**Figure 17:**
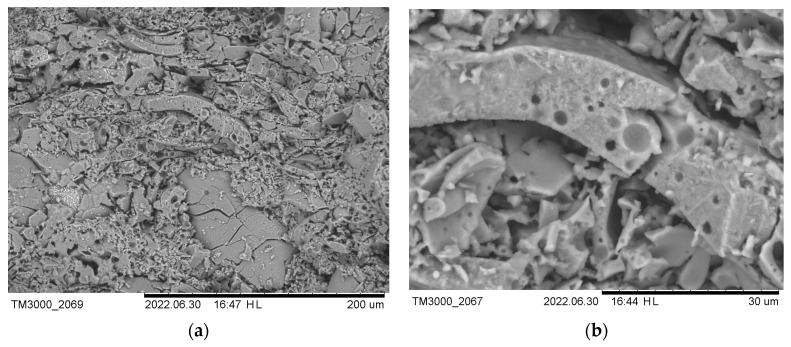
At a temperature of 1200 °C, sintered CS1-0 cenospheres (CP 250 bar) at ×300 (**a**) and ×1000 (**b**) times magnification.

**Figure 18:**
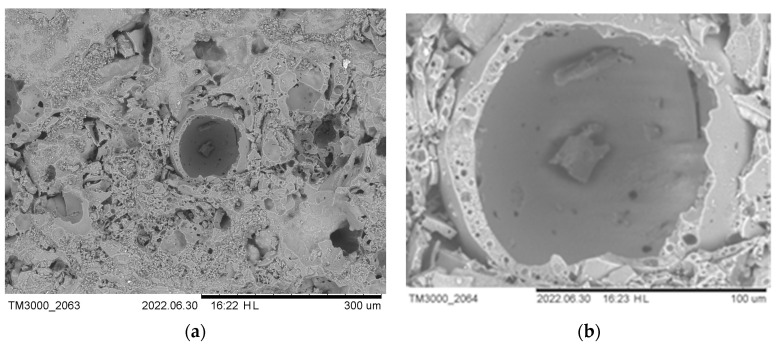
At a temperature of 1300 °C, sintered CS1-0 cenospheres (CP 25 bar) at ×200 (**a**) and ×800 (**b**) times magnification.

Scanning electron microscopy is used to study the structure of materials. To test the strength of the cenospheres, individual samples were pressed at a higher pressure (250 bars). SEM images (Figure 16 and Figure 17) show that the cenospheres are either partially or fully crushed in the case of pure cenosphere ceramics. The formation of mullite needles on the surface of the cenospheres can be observed in the SEM images of the sintered samples of cenospheres (CS1 and CS2) (Figure 16). At a CP of 250 bars, almost all spheres are destroyed (Figure 17). In well-sintered (1300 °C) CS1 samples at low CPs (25–50 bar), many cenospheres are intact (Figure 18).

**Figure 19:**
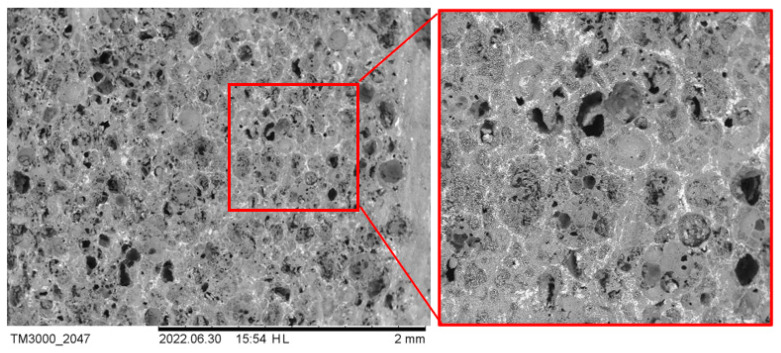
Microstructure of ceramic CS1–20 sintered at 1300 °C (CP 25 bar).

**Figure 20:**
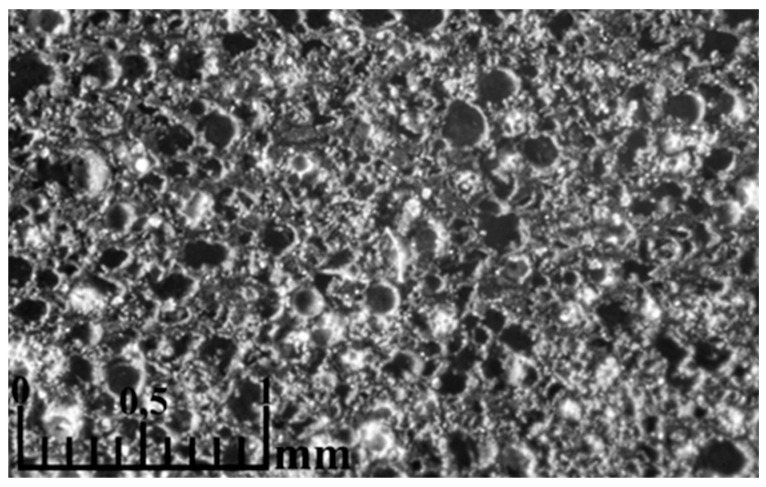
Microstructure of ceramic CS1-20 sintered at 1300 °C (CP 25 bar).

The addition of CoFe_2_O_4_ improves sintering and mechanical strength, so the spheres are less crushed (Figure 19). In compositions of cenospheres with CoFe_2_O_4_, most of the spheres are healthy (Figure 20 (the image was taken with an optical microscope)). Ferrite particles cover the cenospheres, thus increasing the density of the material and improving its mechanical properties.

From Figure 5, Figure 6, Figure 7, Figure 8, Figure 9, Figure 10, Figure 11 and Figure 12, it can be seen that samples sintered from raw materials of different particle sizes have a huge gap in compressive strength when other conditions remain unchanged. For example, in Figure 8 and Figure 9, the compressive strength difference between CS1-7.5 and CS2-7.5 samples sintered at 1300 °C is nearly 30 MPa (CP = 125). This effect could be explained by the synergy of a few phenomena.

**Figure 21:**
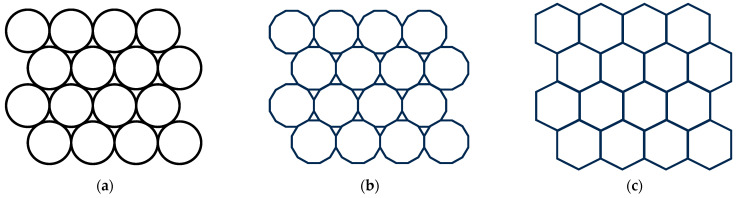
CS sintering main stages: the initial stage (**a**), the start of deformation (**b**), and maximally deformed–tightly compacted (**c**).

First, as established in a previous work [15], materials CS1 and CS2 have similar sintering behaviours until 1250 °C (Figure 6). At temperatures higher than 1250 °C, higher shrinkage (sintering) occurs. Common possible sintering behaviour is schematically illustrated in a diagram (Figure 21a). According to our observation of the sintered CS1 and CS2, we propose distinguishing three main compaction stages during the sintering process. The first initial stage, Figure 21a, in which CSs are undeformed and have small spot contact points; in the second stage (Figure 21b), deformation starts for both CS1 and CS2 at 1250 °C, and contact point spots increase; the third stage with maximal deformation, tightly compacted but not melted, is noted at 1300 °C (Figure 21c), so the first factor playing a role in the increased compression strength is temperature (at 1300 °C).

**Figure 22:**
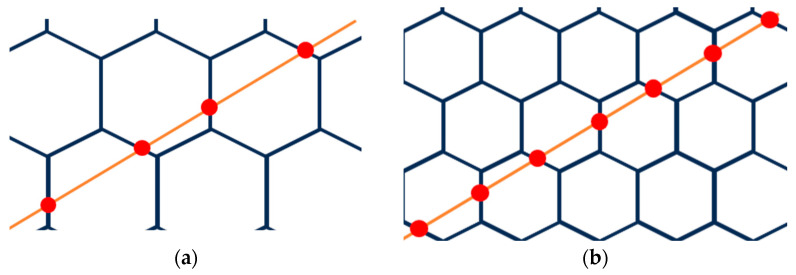
Schematic representation of tightly compacted CS1 with particle size 150–250 μm (**a**) and tightly compacted CS2 with particle size 63–150 μm (**b**).

Second, there is a difference between the particle sizes of CS1 and CS2. Schematic microstructures of tightly compacted CS1 and CS2 with different particles are represented in Figure 22. A randomly oriented orange line imitates a cross-section in the case of breaking under a load. The red dots illustrate the SC walls’ breaking points under the load. As can be seen, in the case of CS2 (Figure 22b), almost double number of the walls should be broken in the case of breaking under a load for CS2 in comparison with CS1 because of the smaller particle size. As a result, for this, it is necessary to spend more energy; this is the second input to the increased compression strength—the role of particle size.

**Figure 23:**
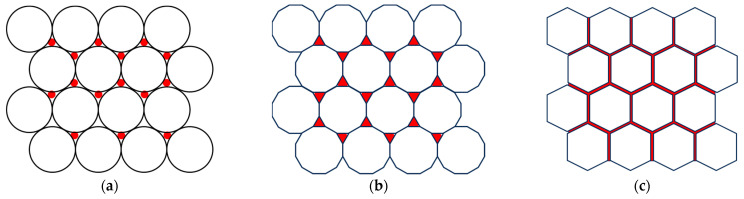
Schematic illustration of ferrite distribution in CS-ferrite composite during sintering main stages: in the initial stage (**a**), at the start of deformation (**b**), and when maximally deformed (**c**).

The third factor is the amount and distribution of the ferrite nano-additive. We propose a possible explanation illustrated in Figure 23, in which it can be seen that the nano-additive is homogenously distributed between SCs at different sintering stages. A uniform distribution of the nano-additive between CSs (Figure 19) at the third sintering stage ensures better sintering due to the cohesion of nanoparticles and promotes sintering as well as overall mechanical strength. The third input to the increased compression strength is the presence of the nano-additive at a concentration of 7.5%, which is close to the optimal concentration (as per compression strength evaluation criteria).

As a result of this research, the optimal CP for samples of the given size is 25–75 bar.

The mechanical properties (compressive and bending strength) of some samples depending on the CP, sintering temperature, and concentration of ferrites are given in Table 2. For this purpose, plates measuring 65 × 65 × 9 mm were made from which samples were cut to determine compressive strength (10 × 10 × 9 mm) and bending strength (10 × 9 × 60 mm).

As shown in Table 2, a geometric factor can be observed—the density of cylindrical tablets obtained under the same conditions (temperature, CP) is higher than that of large plates. Compared to small-sized samples (in our case, the surface area of the small samples perpendicular to the compaction direction was 1 cm^2^), the surface area of the plates exceeds 40 cm^2^. Therefore, in a small volume, the material compresses more, and the initial density at the same CP is higher.

**Figure 24:**
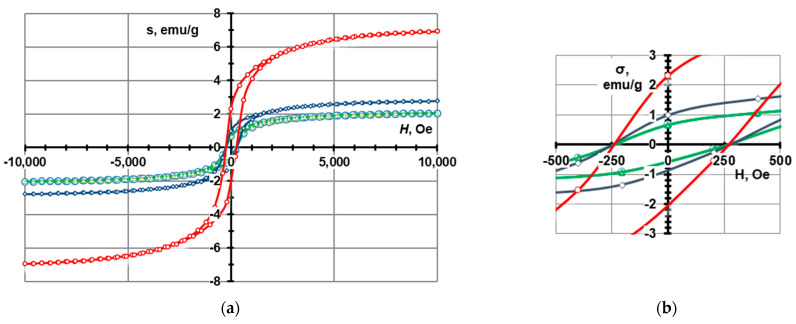
Magnetization hysteresis of CS1 nanocomposites with CoFe_2_O_4_ nanoparticles sintered at 1200 °C. The curve in red corresponds to 7.5, dark grey corresponds to 10 and red corresponds to 20% of CoFe_2_O_4_ content, ranging (**a**) from −100,00 to 10,000 *H*, Oe and (**b**) from −500 to 500 *H*, Oe.

Figure 24 shows hysteresis loop magnetisation (*σ*) as a function of the applied magnetic field (*H*) for the three CS1 samples sintered at 1200 °C. Values of the saturation magnetisation *M*_s_, remanent magnetisation *M*_r_, and coercivity *H*_c_ are listed in Table 3. The coercivity (*H*_c_) of the ceramics with various ferrite contents was 275 Oe. But as the ferrite content increases, the saturation magnetization (*M*_s_) and remnant magnetisation (*M*_r_) of the ceramics also increase. The *M*_s_ values for ceramics with 7.5, 10, and 20% ferrite were 6.93, 2.78, and 2.04 emu/g, respectively, and the *M*_r_ values were 2.3, 1.0, and 0.65 Oe, respectively. This shows that the soft magnetic properties are improved with increasing ferrite content. Soft magnetism is indicative of low volume resistivity and better EMI shielding, as observed in FeCoNi-coated carbon fibres [55] and CI/Ti3C2Tx/PVDF multilayer structured composite films [56]. 

The coercivity of a material dictates its magnetization capacity. If it is high, the material stays magnetized for long and vice versa. For CoFe_2_O_4_ nanoparticles the coercivity was reported to be 188.57 Oe at room temperature [57]. Compared to pure CoFe_2_O_4_, the ceramic and ferrite material studied in this study had a higher value of coercivity of 275 Oe, which suggests that some hardening in the ferromagnetic properties of the material is detected. Although it is important to note that while for CS1 ferrite sintered at 1200 °C, the coercivity is the same, it is higher for CS2 at the same sintering temperature. Typically, this is not the case the higher the coercivity of the material.

**Figure 25:**
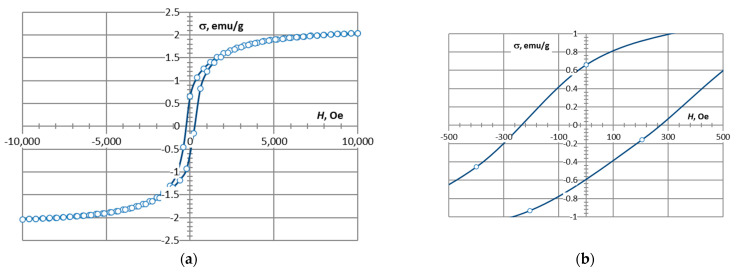
Magnetization hysteresis of CS2 nanocomposites with 7.5 wt.% CoFe_2_O_4_ nanoparticles sintered at 1200 °C, ranging (**a**) from −10,000 to 10,000 *H*, Oe and (**b**) from −0.5 to 0.5 *H*, Oe.

The electromagnetic properties (saturation magnetization *M*_s_, remanent magnetization *M*_r_, and coercivity *H*_c_) of the material increase in direct proportion to the concentration of ferrite nanoparticles in the material (Table 3, Figure 24 and Figure 25).

Increasing the CP and sintering temperature contributes to the sintering of the material and the increase in mechanical strength, both in the case of pure cenosphere ceramics and in the composition with CoFe_2_O_4_. As the images obtained using an SEM show, in the case of pure cenosphere ceramics, the cenospheres are partially or destroyed, depending on the CP. Low CPs should be used to preserve the closed pore structure as much as possible, but then the mechanical strength of the material is not great. The addition of CoFe_2_O_4_ nanoparticles significantly improves the sinterability of ceramics even at low CPs, allowing them to obtain highly porous ceramics with good mechanical strength. An X-ray phase analysis (Figure 15) shows that in the case of cenosphere—CoFe_2_O_4_ ceramics, there is a slight interaction between them—the mullite partially decomposes and Al_2_O_3_ enters the structure of CoFe_2_O_4_, while SiO_2_ precipitates as a separate phase. 

The electromagnetic properties of the material are enhanced in direct proportion to the concentration of ferrite nanoparticles in the material.

**Figure 26:**
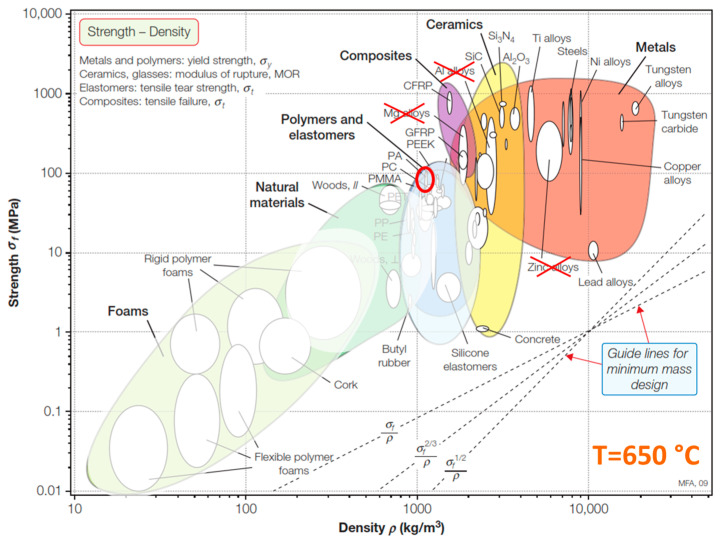
Material class map in the strength–density coordinates, adopted from [58].

M Ashby proposed evaluating materials by simultaneously evaluating various properties [58]. However, the original diagram is valid in a very narrow temperature region—until the material is not melted, burned or decomposed. Figure 26 represents a modified M. Ashby material class map in strength–density coordinates at temperature of 650 °C. As can be seen, classes of materials such as polymers and natural materials (wood) should be excluded from the diagram due to their melting, burning, and decomposition (faded colours). Also, materials with aluminium and magnesium matrix bases also lose mechanical stability due to their melting (crossed out in red). As can be seen, these material classes are the majority of light-weight materials.

The red oval in Figure 26 represents the high-temperature, lightweight ceramics with nano-sized ferrites discussed in this work. As described in detail in [28], almost all lightweight materials for EMI shielding are based on a polymer matrix or have very low mechanical properties. The CS-ferrite composites discussed in this work simultaneously have a low density and significant mechanical properties, accomplishing a potential EMI shielding effect which could find applications in various devices in the aerospace and defence sectors. In Table 4, lightweight composite materials suitable for EMI shielding are represented, grouped by material types: polymer/organic materials, carbon foams, metals and alloys, ceramics, and the material developed in this study.

As can be seen in Table 4 and as also supported by the M. Ashby diagram (Figure 26), materials with low density values (polymer/organic materials and carbon foams) and materials with the lowest density have the lowest mechanical properties. Additionally, as was mentioned above, they have the lowest temperatures of integrity (250–350 °C). Materials containing carbon in their structure—carbon foams and aerogels—have good enough EMI SE. However, they have low mechanical properties and also a low temperature integrity until 450 °C. The next material class—composites with a metal (Al) matrix—has high enough mechanical properties (up to 176 MPa) and density ranging from 1.30 to 1.52 g·cm^–3^. However, these types of materials maintain their integrity up to 660 °C (the melting point of Al). Ceramic materials have the closest the mechanical properties and density. Hierarchical porous SiC-NW-Si_3_N_4_ has a higher density (up to 25%) but at least 300–400% regarding mechanical property values (up to 240 MPa). However, compared to the CS-CoFe_2_O_4_ composites studied (which have mainly closed pores) in this research, SiC-NW-Si_3_N_4_, characterised by mainly open pores, is permeable for gases and liquids, which could be an issue in the case of its use in open air. To demonstrate EMI SE stability in time under open-air and high-temperature exposure conditions, the EMI SE of CS–CoFe_2_O_4_ composites must be determined in the future, not only in an “as prepared” condition but also after exposure to heating and fresh and salt water. 

## 4. Conclusions

A lightweight, high-temperature ceramic material with a ferromagnetic shielding effect was obtained via a conventional pressure-sintering method using cenospheres and CoFe_2_O_4_ nanoparticles. Additives of ferrite nanoparticles improved the sintering of the cenospheres, increasing the material’s density and mechanical properties. The porous structure of the samples largely depends on the CP of the samples: at CPs of 25–75 bars, whole cenospheres are primarily preserved in the sample; at higher CPs, there is significant crushing of cenospheres, a decrease in porosity, and an increase in density. Depending on the sample CP and sintering temperature, samples with a density of 1.2–2.1 g/cm^3^ and a compressive strength of 1.5–125.0 MPa were obtained. The bending strength of the obtained samples was determined within 10–20 MPa. The magnetic properties of the samples change in proportion to the amount of CoFe_2_O_4_ in the sample.

## Figures and Tables

**Table 1:** Magnetic properties of CoFe_2_O_4_.

Ferrite	Magnetic Properties		
	*M*_s_, emu/g	*M*_r_, emu/g	*H*_c_, Oe
CoFe_2_O_4_	75.4	32.0	780

**Table 2:** Density and compressive and bending strength of samples CS1 and CS2 with different contents of CoFe_2_O_4_. Pressure, 240 bar; sintered at 1300 °C.

Sample	Density, g·cm^–3^ (%)	Water Absorption (W), %	Apparent Porosity (π), %	Compressive Strength (σ_comp._), MPa	Bending Strength (σ_bend._), MPa	Flexural Modulus, MPa	Deformation (ε), %
CS1-0	1.03 (32.2)	50.7	52.0	12.4 ± 0.1	7.1 ± 0.4	1960 ± 180	0.39
CS1-7.5	1.17 (35.0)	45.9	49.9	29.3 ± 2.8	10.2 ± 0.3	5190 ± 130	0.41
CS1-10	1.31 (38.4)	33.7	44.2	47.4 ± 12.6	17.9 ± 1.6	4830 ± 450	0.52
CS2-0	1.05 (32.8)	48.8	51.3	17.4 ± 5.8	6.0 ± 0.4	2690 ± 310	0.21
CS2-10	1.32 (38.9)	34.2	45.1	41.7 ± 6.2	12.3 ± 1.9	5220 ± 490	0.23

**Table 3:** Magnetic properties of CS1 and CS2-CoFe2O4 ceramics sintered at 1200 °C.

Sample	Magnetic Properties		
	*M*_s_, emu/g	*M*_r_, emu/g	*H*_c_, Oe
CS2-7.5 wt.% CoFe_2_O_4_	1.17	0.47	375
CS1-7.5 wt.% CoFe_2_O_4_	2.04	0.65	275
CS1-10 wt.% CoFe_2_O_4_	2.78	1.00	275
CS1-20 wt.% CoFe_2_O_4_	6.93	2.30	275

**Table 4:** Lightweight composite materials suitable for EMI shielding, grouped by material types: polymer/organic materials, carbon foams, metals and alloys, ceramics, and the material developed in this study.

Material Type	Density (g·cm^–3^)	Compression Strength (MPa)	Band (GHz), EMI SE (dB), or Other	Ref.
**Polymer/organic materials**				
CNT + mesoporous carbon micro HS + water-based PU + polyvinyl alcohol	0.23–0.26	3	Band 8–12 GHz EMI SE 28 dB	[59]
CoFe_2_O_4_/Polyetherimide composites	N/A	N/A	Saturation and remanent magnetization values are from 4.39 to 27.9 and 1.47–9.57 emu·g^−1^	[60]
**Carbon foams**				
Ag@C-1000	0.00382	N/A	Band 8.2–12.4 GHz EMI SE 70.1 dB	[61]
Cu/large flake size graphene film		N/A	Band 1–2 GHz EMI SE 61.39–63.29 dB	[61]
CuNW@G core–shell aerogels	0.165	N/A	Band 8–18 GHz EMI SE 52.5 dB	[61]
Carbon/MnO_2_ foam		N/A	Band 8.2–12.4 GHz EMI SE 50 dB	[61]
Carbon/GO/SiO_2_		N/A	Band 8.2–12.4 GHz EMI SE 24 dB	[61]
**Metals and alloy composites**				
Al foams	0.35 to 0.71	17.5	N/A	[62]
AlSi7 foams	0.35 and 0.66		N/A	[62]
AZ61-CS	0.9–1.1	16–30	N/A	[63]
AZ61 with spherical carbamide granules	0.79–1.02	16–28	N/A	[64]
Al99.5–SL150 with ceramic microballoons	1.43	169	N/A	[64]
Al99.5–SL300	1.52	154	N/A	[64]
AlSi12–SL300	1.37	176	N/A	[6]
Opened-cell porous aluminium foam	N/A	N/A	Band 1–8 GHz EMI SE 40–70 dB	[61]
Aluminium foam	N/A	N/A	Band 130–1800 MHz EMI SE 2575 dB	[61]
CNTs/Cu foams	N/A	N/A	Band 8.2–12.4 GHz EMI SE 33.63 dB	[61]
**Ceramics**				
Al_2_O_3_/SiO_2_	N/A	24	Band 2–18 GHz EMI SE–55 dB	[65]
Hierarchical porous SiC-NW-Si_3_N_4_	Bulk density 1.61–1.92	142.19–240.36	Good microwave absorption properties	[66]
Fe3O4 particles coated with Al	N/A	N/A	Band 5–13 GHz EMI SE from −16.2 to −12.5 dB	[67]
Cu–Ni–CNT foam	0.24	N/A	Band 8–12 GHz EMI SE 47.5 dB	[61]
**This research**				
CS–CoFe_2_O_4_ * 7.5 wt.%, sintered 1100 °C	1.33–1.65	40–65	Saturation and remanent magnetization values are from 2.04 emu·g^−1^	---
CS–CoFe_2_O_4_ * 7.5 wt.%, sintered 1200 °C	1.35–1.67	27–44	Saturation and remanent magnetization values are 1.17 emu·g^−1^	---
CS–CoFe_2_O_4_ * 20.0 wt.%, sintered 1200 °C	1.54–1.9	58–98	Saturation and remanent magnetization values are 6.93 emu·g^−1^	---
CS–CoFe_2_O_4_ * 7.5 wt.%, sintered 1300 °C	1.59–1.98	76–106	Saturation and remanent magnetization values are from 2.04 emu·g^−1^	---

HS—hollow sphere; LC—low carbon; PU—Polyurethane; CNT—carbon nanotubes; EMI SE—electromagnetic shielding effectiveness; NEW—nanowire; *—nano-size.

## Data Availability

Data are contained within the article.
